# HPV infection, anal intra-epithelial neoplasia (AIN) and anal cancer: current issues

**DOI:** 10.1186/1471-2407-12-398

**Published:** 2012-09-08

**Authors:** Margaret A Stanley, David M Winder, Jane C Sterling, Peter KC Goon

**Affiliations:** 1 Department of Pathology, University of Cambridge, Tennis Court Road, Cambridge, CB2 1QP, UK

**Keywords:** Anal cancer, Anal intraepithelial neoplasia, AIN, Progression, Human papillomavirus

## Abstract

**Background:**

Human papillomavirus (HPV) is well known as the major etiological agent for ano-genital cancer. In contrast to cervical cancer, anal cancer is uncommon, but is increasing steadily in the community over the last few decades. However, it has undergone an exponential rise in the men who have sex with men (MSM) and HIV + groups. HIV + MSM in particular, have anal cancer incidences about three times that of the highest worldwide reported cervical cancer incidences.

**Discussion:**

There has therefore traditionally been a lack of data from studies focused on heterosexual men and non-HIV + women. There is also less evidence reporting on the putative precursor lesion to anal cancer (AIN – anal intraepithelial neoplasia), when compared to cervical cancer and CIN (cervical intraepithelial neoplasia). This review summarises the available biological and epidemiological evidence for HPV in the anal site and the pathogenesis of AIN and anal cancer amongst traditionally non-high risk groups.

**Summary:**

There is strong evidence to conclude that high-grade AIN is a precursor to anal cancer, and some data on the progression of AIN to invasive cancer.

## Background

Approximately 15 of the 30–40 human papillomavirus (HPV) types infecting the ano-genital mucosae (cervix, vagina, vulva, anus and penis) are oncogenic viruses causing ~ 5.2% of total human cancers [1]. Like cervical cancer, squamous cell carcinoma of the anus (anal SCC) is thought to be preceded by a spectrum of intra-epithelial changes, anal intra-epithelial neoplasia (AIN), of varying cytological and histological severity AIN 1 - mild, AIN 2 - moderate, AIN 3 - severe, similar to cervical intra-epithelial neoplasia (CIN), that correspond to the CIN spectrum CIN 1, CIN 2/3 [2-4]. The success of current HPV L1 prophylactic vaccines in preventing high-grade dysplasia of the cervix (and consequently the end-result of cancer) has raised the questions of whether these vaccines will be successful in preventing other HPV-related cancers, and whether subsequent licensing of vaccine for non-cervical cancers is merited.

In this context, we will summarise the available evidence that seeks to answer three questions which are currently discussed for anal cancer and pre-cancers.

 1) The lack of data on AIN and anal cancer from the larger percentage of the community i.e. heterosexual males and non-HIV-positive or non-high risk women, which has meant that the majority of the inferences are therefore extrapolated from MSM data.

 2) That AIN is a precursor lesion to invasive anal cancer.

 3) The rate of progression of AIN 3 to invasive anal cancer.

## Discussion

### Epidemiology and biology

Anal SCC is an uncommon cancer but the incidence worldwide is increasing in men and women [3]. Women have a higher incidence rate in age groups greater than 50 years but men dominate the 20–49 year old age group. In men and women, common risk factors are receptive anal sex, lifetime number of sexual partners, a history of genital warts and cigarette smoking. Further, in women, additional risk factors are a history of high grade vulvar intraepithelial neoplasia (VIN 3), cervical intraepithelial cancer (CIN 3), vulvar cancer or cervical cancer [4]. Immunosuppressed individuals, such as solid organ transplant recipients and HIV-infected patients, are also more susceptible to HPV-related cancers [5,6]. Most anal SCCs are a consequence of sexually or otherwise acquired infection in the anal mucosa with oncogenic HPV subtypes, predominantly HPV 16 [7].

In Scotland in the period 1975 to 2002 the incidence of anal SCC doubled to 0.37 per 100,000 in men and 0.55 in women [8]. Data from the National Survey of Sexual Attitudes and Lifestyles (NATSAL) in 2000 (quoted in [8]) indicates that in Scotland reported prevalence of anal intercourse in women (11.16%) was substantially higher than men (0.7%), indicating that this behaviour may partially account for the observed difference in prevalence of anal cancer between the genders. Furthermore, the anatomic proximity of the vaginal introitus to the anus also facilitates non-sexual and auto-inoculation in women via vaginal secretions, digital or fomite transference [9,10]. The proportion of Scottish women reporting the practice of anal intercourse is significantly lower to similar studies in the U.S., where a prevalence of 22 – 29% has been documented [11,12]. These discrepancies may represent true population behavioural differences or, perhaps more likely, under-reporting. Even anonymised studies may show significant bias when reporting behavioural aspects, especially when studies are smaller. The overall risk for anal SCC is linked to sexual behaviour between or within genders, not sexual preference; increases in incidence were shown before the advent of the HIV epidemic [3]. The majority of studies of prevalence of anal HPV infection and AIN have been in the MSM cohort, especially HIV-positive MSM, as this is the highest risk group [13], but there are also some data from high-risk HIV-positive and HIV-negative women [14]. However, the paucity of data from the heterosexual male and non-HIV-positive or non-high risk female populations (largest sections of the general population who make up a substantial number of anal cancer patients) has been a problem in the clinical management of these patients, particularly in determining the rationale and feasibility of instituting a screening program. Nevertheless, it is likely that among men, MSM account for a disproportionate amount of anal intercourse and anal cancer.

Recent emerging data suggests that anal HPV (including intra-anal and peri-anal HPV) infection is common. Nyitray et al. 2008 [15] reported that the prevalence of HPV DNA, detected in 222 heterosexual men, was 16.6% for the anal canal and 21.3% for the perianal area. Of the patients with anal HPV infection, fully 33.3% had an oncogenic high-risk HR-HPV type (as defined by work from cervical cancer and its precursors). A recent follow-up study by the same group, on men from Brazil, Mexico and the USA has extended these findings [16]. In comparing only HIV-negative men (1305 heterosexual versus 176 homosexual), they found anal canal HPV prevalence of 12.2% and 47.2% in these groups respectively. Two previous studies examining heterosexual men i.e. Van Doornum et al. 1994 [17], and Nicolau et al. 2005 [18], had reported anal HPV DNA prevalences of 35% from 85 men and 8% of men, respectively. The large discrepancies seen are most likely due to differences in technique, sample collection, different cohorts and, in particular, the use of currently available highly sensitive PCR and line blot assays with amplification steps.

Anal HPV infection data in HIV-negative women is slightly better documented. The best available data comes from the Hawaii HPV Cohort study; Hernandez et al. 2005 [19] reported a baseline prevalence of 27% anal HPV infection in this cohort. The same group has now reported that during an average follow-up period of 1.3 years, 70% of the women developed incident anal HPV infection [20]. Moscicki et al. 1999 [9] reported that 66.7% of women with abnormal anal cytology had detectable HPV compared to 12.7% of women with normal anal cytology. Finally, Palefsky’s group [14] reported a prevalence of 42% in a cohort of HIV-negative women with high-risk behaviour. The evidence is therefore slowly accumulating that infection of the anal region with HPV in both heterosexual men and non-HIV infected women is relatively common.

Natural history studies and data on the role of persistent anal infection with HPV in both sexes are considerably less compared to that in women for cervical cancer. The largest ongoing longitudinal study in women is that by the investigators of the Hawaii HPV cohort where they showed that in 431 women (followed at 4-monthly intervals), 50% had an incident anal infection during a mean duration of 1.2 years [21]. The median duration for infection with a high-risk subtype was 150 days. This group has further demonstrated that HPV anal infection was likely to be sequentially acquired after initial cervical infection, and that anal infection is cleared in 87% of women within 1 year [22]. This is shorter than the generally reported rate for the cervix (~90% cleared by 2 years) [23]. Interestingly, one study showed that 6 month persistence of HPV 16 was absent in heterosexual men, whereas it occurred in 5.1% of MSM patients [24].

These data may partially explain the finding that anal cancer incidence is much lower than that for cervical cancer if we accept the hypothesis that persistent infection with HPV is also required for anal cancer development. High grade AIN (HGAIN) can be identified using high-resolution anoscopy (HRA) or colposcopy of the anus and perianus after application of 3-5% acetic acid. However, in comparison to the number of cervical colposcopists, there are currently far fewer trained experts in HRA and this partly explains why there is even less data published on the prevalence of HGAIN in the general population. Since there are no recommendations for screening, many patients are either diagnosed serendipitously during surgery for benign anal conditions or occasionally during colonoscopy or if they present with anal symptoms [25,26].

High grade intra-epithelial neoplasia at all ano-genital sites (anus, cervix, vagina, vulva, penis) is likely to be a precursor to invasive cancer at that site but the potential for progression is best documented in the cervix as a consequence of a large unethical study in which, during the period 1955–1976, definitive treatment of CIN 3 was withheld from a substantial cohort of New Zealand women. The cumulative incidence of cancer of the cervix or vaginal vault in this cohort was 31.3% at 30 years as compared with 0.7% receiving adequate treatment [27]. Clearly such a study cannot be duplicated at other sites but progression of AIN to anal SCC has been assessed in small studies with surveillance and treatment, but with a relatively short follow up of 5–10 years. Two studies conducted on HIV-negative cohorts stand out. Scholefield’s group [28] has published on a cohort of 35 HIV-negative patients (26 women and 9 men) with AIN 3 and followed up for a median duration of 63 months (range 14 – 120 months). The median age was 43 (range 31 – 62 years). Three of these 35 patients (8.5%) developed invasive carcinoma at the sites of previous AIN 3 disease and all three were systemically immunosuppressed from long term oral therapy. The authors suggest that immunosuppressed patients (6 in this study – all from long term oral therapy, such as corticosteroids, for various conditions) and those with multifocal AIN (7 in this study) are at higher risk of malignant transformation. This rate of malignant conversion is not too dissimilar to that seen in VIN 3 conversion to vulvar cancer (~9% over 5 years) [29].

Another important study has reported on a cohort of 72 patients with AIN, followed for a median of 60 months (range 18–112 months) and with a median age of 49 years (range 18 – 81 years) [30]. 8 patients progressed to invasive SCC, 2 from AIN 2 (10 patients) and 6 from AIN 3 (45 patients). Not one of 17 patients with AIN 1 progressed to cancer during the follow-up period. Twenty-five patients downgraded their lesions. The authors suggest that since 13% of their AIN 3 patients converted to cancer during the follow-up period, it is necessary to keep all AIN 3 under close surveillance. However, the data from these 2 small studies show that even with surveillance and treatment, on average 9-13% AIN 3 may progress to invasive disease over 5–10 years, comparable to that observed for progression of CIN 3 over similar time periods.

However, a recent meta-analysis has indicated that progression rates from HGAIN to anal cancer are approximately one in 600 per year in HIV-positive MSM and one in 4000 per year in HIV-negative MSM patients [31], substantially lower than the one in 80 per year observed in comparable cervical disease [27]. One possible explanation for this discrepancy is that these estimates were based on multiple cross-sectional studies and pooled prevalence rates of HGAIN and anal cancer incidence rates, rather than prospective follow-up studies. However, it cannot be excluded that HGAIN might regress more often than CIN 3, perhaps being partially attributable to the fact that HGAIN encompasses both AIN 2 and AIN 3, with AIN 2 probably containing a mixture of HG and LG HPV types.

## Summary

There are strong supportive data for the contention that HGAIN is a precursor lesion to invasive anal carcinoma. However, there are limited data on the prevalence of HGAIN in the general population. There are also some data giving estimates of the natural progression of AIN into invasive cancer over different follow-up periods. Finally, there are current and very recent studies on anal HPV infection in traditionally non-high risk groups continuing to emerge, which help to avoid mere extrapolation of data from high-risk groups into the general community. There is now a need for multi-centre prospective studies on progression of AIN 3 to cancer and on the effectiveness of treatment to reduce the incidence of anal cancer.

## Abbreviations

HPV: human papillomavirus; HIV: human immunodeficiency virus; CIN: cervical intra-epithelial neoplasia; AIN: anal intra-epithelial neoplasia; MSM: men who have sex with men; VIN: vulvar intraepithelial neoplasia; OR: odds ratio; RR: relative risk; SCC: squamous cell carcinoma.

## Competing interests

MAS acts as consultant to Sanofi Pasteur-MSD, Lyon, France and MAS and PKCG have previously received an educational grant from Sanofi-Pasteur MSD. MAS also acts as consultant to Merck Research Laboratories, Westpoint, USA, and GSK Biologicals, Rixensart, Belgium. This work was supported by grants from the British Skin Foundation (ref: 806), Addenbrooke’s Charities Trust (ref: 25/09CB) and Cancer Research UK (ref: C26262/A8633) to PKCG.

## Authors’ contributions

All authors contributed significantly to the work and have seen and approved the final content of the article.

## Pre-publication history

The pre-publication history for this paper can be accessed here:

http://www.biomedcentral.com/1471-2407/12/398/prepub
